# EXAMINING TWO CONSECUTIVE YEARS OF OLDER ADULTS’ EVERYDAY PROSPECTIVE MEMORY LAPSES AND INFLAMMATION BY GENDER

**DOI:** 10.1093/geroni/igad104.3322

**Published:** 2023-12-21

**Authors:** Erin Harrington, Jennifer Graham-Engeland, Martin Sliwinski, Jacqueline Mogle, Richard Lipton, Mindy Katz, Christopher Engeland

**Affiliations:** University of Wyoming, Laramie, Wyoming, United States; Pennsylvania State University, University Park, Pennsylvania, United States; Pennsylvania State University, University Park, Pennsylvania, United States; Clemson University, Clemson, South Carolina, United States; Albert Einstein College of Medicine, Bronx, New York, United States; Albert Einstein College of Medicine, Bronx, New York, United States; Pennsylvania State University, University Park, Pennsylvania, United States

## Abstract

Prospective memory (PM, i.e., memory for future intention) is vital to daily life. Although PM deficits can distinguish healthy older adults from those in early stages of dementia, limited work has examined PM and biological markers associated with memory decline. Extending past work, we examined self-reported PM lapses in daily life and systemic (circulating) inflammatory markers by gender across two years. Older dementia-free adults (n=90, Mage=76.84 years; 62%women) enrolled in the ongoing Einstein Aging Study completed two-waves of data collection (approximately one year apart), each including two-week measurement bursts of daily self-reported PM lapses which were summed across respective bursts. Averaged levels of systemic inflammatory cytokines (IL-1β, IL-4, IL-6, IL-8, IL-10, TNF-a) and C-reactive protein were derived from two blood samples per wave (pre/post burst). Among men, cross-sectional and cross-lagged correlations between PM and IL-8 were significant, such that reporting more PM lapses correlated with higher levels of IL-8 (ps<.009). Among women, wave-1 PM correlated with wave-2 TNF-a (p=.018), and wave-2 PM correlated with wave-2 IL-10 (p=.041). No other links emerged. Consistent with past research, these preliminary findings suggest that IL-8 may be an important correlate of poor daily PM in men. The neural substrate of this association could include accelerated aging or increases in Alzheimer’s or vascular pathology. Among women, lagged relationships between PM and inflammation may be indicative of pathology. Continued investigation into PM and inflammation that account for gender may reveal longitudinal links that could aid in early identification of persons at risk for cognitive decline.

